# Analysis of disease progression in patients with transthyretin cardiac amyloidosis

**DOI:** 10.1186/1750-1172-10-S1-O10

**Published:** 2015-11-02

**Authors:** Julian D Gilmore, Mathew S Maurer, John Vest, Verena Karsten, Christine Powell, Andrew Strahs, Jared Gollob, Philip N Hawkins

**Affiliations:** 1 University College London Medical School, National Amyloidosis Center, NW32PF, London, UK; 2 NewYork-Presbyterian/Columbia, Clinical Cardiovascular Research Lab for the Elderly, 10034, New York, NY, USA; 3 Alnylam Pharmacuetical, Clinical Development, 02142, Cambridge, MA, USA

## Background

In transthyretin (TTR) cardiac amyloidosis, myocardial deposition of liver-derived TTR fibrils results in heart failure and death. Wild-type TTR (ATTRwt) amyloidosis is an acquired disease, whereas familial amyloidotic cardiomyopathy (FAC) is a hereditary form of the disease caused by mutations in the TTR gene resulting in the deposition of both mutant and wild-type TTR. Published data on disease progression in TTR cardiac amyloidosis is limited. To explore this further, we analyzed natural history data in ATTRwt amyloidosis patients to characterize disease progression in this population and compared this to our previously reported data in FAC.

## Methods

Demographic and overall survival (OS) data from patients with ATTRwt amyloidosis (ATTRwt OS cohort) was collected retrospectively from two centers. Six-minute walk distance (6MWD) data from patients with ATTRwt amyloidosis (ATTRwt 6MWD cohort) was collected prospectively from a single center. Data from ATTRwt amyloidosis patients was compared descriptively with our previously reported results from FAC patients collected at the same centers.

## Results

The ATTRwt OS cohort (N=255, median age 78 years, 47% NYHA class II, 22% NYHA class III) had a median survival (95% CI) from time of first visit of 31.6 months (23.6, 41.0). The ATTRwt 6MWD cohort (N=153, median age 77 years, 65% NYHA class II, 25% NYHA class III), had a mean baseline 6MWD (+/- SEM) of 313m (10). In these ATTRwt amyloidosis patients, the mean change from baseline in 6MWD (+/- SEM) at 6 (N=125), 12 (N=88) and 18 (N=55) months was -30m (7), -59m (13) and -89m (16) respectively. In our previously reported data in FAC patients (N=137, median age 72 yrs, 40% NYHA class II, 43% NYHA class III), median survival (95% CI) was 34.1 months (28.6, 38.5). For a subset of FAC patients in whom 6MWD data was available (N=39, median age 76 yrs, 59% NYHA class II, 38% NYHA class III), mean baseline 6MWD (+/- SEM) was 281m (20), and mean change from baseline in 6MWD (+/- SEM) was -36m (23), -106m (24) and -140m (39) at 6 (N=32), 12 (N=27) and 18 (N=16) months respectively.

## Conclusion

Survival is limited in both ATTRwt amyloidosis and FAC. Patients with both ATTRwt amyloidosis and FAC demonstrate a decline in functional status over an 18 month time period.

